# A rare cause of acute urinary retention in women: meatal condyloma accuminata, a case report

**DOI:** 10.11604/pamj.2016.24.87.9751

**Published:** 2016-05-27

**Authors:** Onder Cinar, Mustafa Suat Bolat, Ekrem Akdeniz, Necmettin Sahinkaya

**Affiliations:** 1 Department of Urology, Samsun Training and Research Hospital, Samsun, Turkey

**Keywords:** Acute urinary retention, condyloma acuminata, female urethra

## Abstract

Acute urinary retention in women is a rarely seen phenomenon due to pharmacological, neuromuscular, anatomical, functional and infectious causes. Human papillomaviruses causing condyloma acuminata is one of the rarely reported viral infectious cause of acute urinary retention in case reports. A 45-year-old woman with acute urinary retention was found to have a round solid lesion on external urethral meatus. Histopathological examination revealed as condyloma acuminata. Urethral condyloma can be treated by local excision as an effective method for early improvement of voiding function. Even if the genital condyloma can be locally excised, patients should be referred to the gynecologists for cervical cancer screening.

## Introduction

Urethral condyloma is a rare entity that may seldom affect urination and causes urinary retention. In this presentation we reported an external meatal condyloma accuminata which mimicked neoplasm in a female patient causing acute urinary retention.

## Patient and observation

**Figure 1:**
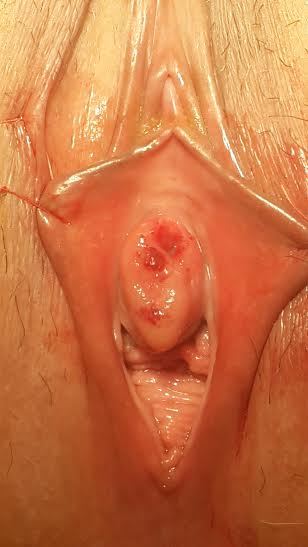
Preoperative image of the lesion surrounding the external urethral meatus

**Figure 2:**
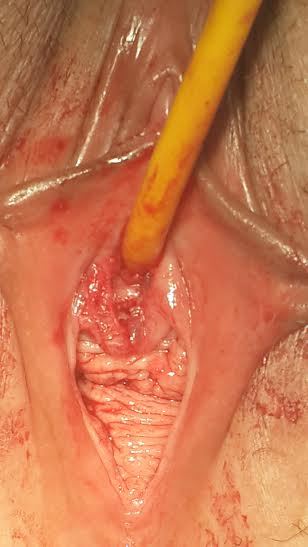
Postoperative image of external urethral meatus

**Figure 3:**
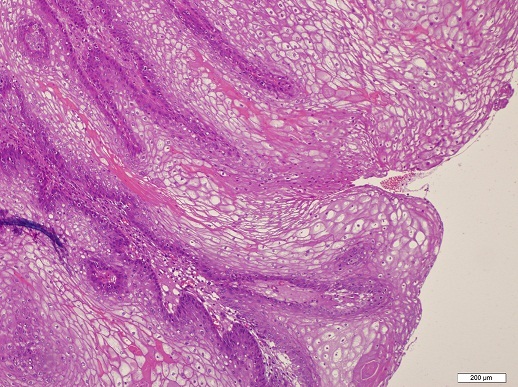
Hyperplasia on the papillary surface and koilocytotic changes in metaplastic squamous epithelial cells

A 45-year-old woman was unable to urinate for the last 24 hours admitted to our clinic with no history of drug addiction, surgery, medication or allergy on her anamnesis. Pelvic examination revealed hyperemic periurethral ulcerated protruded vulvar solid mass surrounding the external urethral meatus (Figure 1). She had difficulty on urination for two weeks. Since she had acute urinary retention, urinary cathaterization was done under sterile conditions and almost 750 ml urine was evacuated with 16 Fr urethral catheter. Urine sample was obtained for microbiological and serological evaluation. After urine culture was proved sterile, the urethral mass was completely excised under spinal anesthesia (Figure 2). Histopathological examination revealed hyperplastic changes on the papillary surface and koilocytotic changes in metaplastic squamous epithelial cells (Figure 3) verifying the human papillomavirus infection. Surgical margins were negative. Urethral catheter was removed two days after the surgery and the patient discharged with normal urination without postvoiding residual urine. At the postoperative first month control, vaginal smear and the type of human papilloma virus was sampled by the gynecologist. Appearence of external meatus was normal and she had no urinary symptoms with no residual volume. Uroflowmetric examination showed normal Qmax and Qaverage values (25.6 ml/s and 12.8 ml/s, respectively).

## Discussion

The incidence of AUR is seldom in women (7 per 100,000) and the male to female ratio is 13:1 in an Scandinavian study [1]. Anatomical and functional causes of bladder outlet obstruction in women include infective and inflammatory reasons such as urethral stricture, urethral caruncle, urinary tract infections or acute vulvovaginitis [2, 3]. In addition; various rare reasons such as herpes zoster infection [4], cytomegalovirus cytitis [5] and eosinophilic cystitis [6] have been described in case reports for acute urinary retention. Meatal involvement of condyloma is reported in%50 of patients [7]. Condyloma acuminata of the urethra is mostly caused by the human papilloma virus serotypes 16 and 18 [8]. Sexually active young adults between the age of 17-33 years are the highest risk group for infection and the incubation period varies from 2 weeks to 6-18 months. The life time number of sexual partners is the most important risk factor identified for genital warts [8, 9]. Symptoms such as split stream, dysuria, urethral bleeding, and infection are seen only%50 percent of the patients. Genital warts may develop as soft, papillary, single, multiple or plaque lesions, representing usually at the genitalia, rectum or urethra. The occurence of AUR due to urethral condyloma is uncommon in both men and women. Treatment methods for external genital condylomas include 5-fluorouracil, interferon, electro coagulation, cryotherapy, photo dynamic therapy, carbon dioxide laser, and local excision. Local reactions such as urethral stenosis, erosion, adhesion and pain may occur as complications. Local excision of the condyloma has better results and is mostly used though electrocoagulation or laser vaporation has higher recurrence rates [10].

## Conclusion

Condyloma acuminata in the female urethra causing acute urinary retention is rarely seen. Urethral benign and malignant lesions should be kept in mind and pelvic examination should be done due to differential diagnosis in women presenting with acute urinary obstruction. This report emphasizes that urethral condylomas causing bladder outlet obstruction can be treated by local excision as an effective treatment method for early improvement of voiding function. Even if the genital condylomas are locally excised patients should be referred to the gynecology clinic for the risk of cervical cancer.
